# LncRNA HIF1A-AS2: a potential biomarker for early diagnosis of acute myocardial infarction and predictor of left ventricular dysfunction

**DOI:** 10.1186/s12872-023-03164-4

**Published:** 2023-03-14

**Authors:** Eman Tayae, Eman Amr, Amr Zaki, Dalal Elkaffash

**Affiliations:** 1 Clinical Pathology Department, Faculty of Medicine, Alexandria University, Alexandria, Egypt; 2 Cardiology Department, Faculty of Medicine, Alexandria University, Alexandria, Egypt

**Keywords:** Long non-coding RNA, Hypoxia-inducible factor 1A, Myocardial infarction, Antisense RNA, Early biomarker

## Abstract

**Background:**

Rapid diagnosis of acute myocardial infarction (AMI) is the subject of many clinical studies as it enables an effective therapy, preventing adverse progression of AMI and increasing survival rates. Recent studies have revealed that specific blood-based long non-coding RNAs (lncRNAs) are deregulated in patients with AMI and serve as promising diagnostic and prognostic tools. The current study aimed to determine the potential role of a hypoxia-responsive lncRNA, hypoxia-inducible factor 1A antisense RNA 2 (HIF1A-AS2), as a biomarker for early diagnosis and predictor of left ventricular dysfunction (LVD).

**Methods:**

This study was carried out on 48 patients with AMI and 50 age-and sex-matched controls. The relative quantification of HIF1A-AS2 expression was done using reverse transcription real‐time polymerase chain reaction.

**Results:**

Compared to the control group, HIF1A-AS2 were significantly higher in MI patients (*P* < 0.001). Interestingly, patients presenting within 3 h of chest pain onset had elevated levels of HIF1A‐AS2 as compared to patients with late presentation. The ROC curve was constructed to assess HIF1A-AS2 as an early marker. It demonstrated higher sensitivity (94%) and specificity (86%). Moreover, the multivariate regression analysis revealed that HIF1A‐AS2 was significantly associated with LVD in the patient group after 6 months follow up (*p* = 0.018).

**Conclusion:**

Our study suggests that HIF1A‐AS2 may be a potential early diagnostic biomarker of AMI with high sensitivity. In addition, it might have a promising role as a predictor of left ventricular dysfunction.

## Introduction

According to the latest WHO Global Health Estimates, cardiovascular diseases, specifically ischemic heart disease (IHD), is still the leading cause of mortality worldwide over the past two decades. However, it currently results in higher increased mortality rates than ever before [1]. Myocardial infarction (MI) is a severe manifestation of IHD. It results from rupture or erosion of a vulnerable atherosclerotic coronary plaque where a totally occluding thrombus leads to Acute ST-segment–elevation MI (STEMI) or from reduced coronary artery blood flow without complete coronary occlusion, coronary artery spasm, coronary embolism results in Non-STEMI (NSTEMI) or unstable angina [2, 3]. Restricted blood supply would lead to irreversible damage of functional cardiomyocytes, ultimately resulting in ventricular failure, with significant adverse effects on life quality and increased mortality [4].

In addition to clinical symptoms and electrocardiographic (ECG) findings, biomarkers levels are the most crucial factor in diagnosing AMI. Cardiac troponin is the diagnostic gold standard for AMI, and its use is recommended by current guidelines [5]. However, exploring new diagnostic and prognostic biomarkers with high sensitivity and specificity, especially for early AMI, could pave the way for the continuous discovery of new therapeutic targets.

Epigenetic processes, defined as heritable changes in gene expression that occur without changes to the DNA sequence, have emerged as a promising area in cardiovascular disease research. Epigenetic biomarkers include DNA methylation, histone modifications, and non-coding RNA mechanisms [6]. Over the past decade, whole transcriptome screening has revealed that nearly the entire human genome is transcribed. However, only a minor fraction (2%) of these transcripts are translated into proteins, while the remaining plethora of transcripts represent ncRNAs [7].

More than 80% of ncRNAs represent a heterogeneous group of long non-coding RNA (lncRNAs) (200 nt–1 kb in length). LncRNAs are significantly more tissue-specific than protein-coding genes and are predominantly localized in the nucleus, whereas mRNA is abundant in the cytoplasm [8]. LncRNAs exert their effects through a variety of mechanisms that include chromatin and chromosome condensation through histone modifications, transcriptional regulation, posttranscriptional regulation, and regulating miRNA expression [9].

In the past few years, many studies have demonstrated that a group of lncRNA expressions is modulated by hypoxia [10]. As MI results from acute and prolonged deficits in oxygen supply, which trigger a series of severe biochemical and metabolic disturbances in the cardiomyocytes [11], we attempted to determine the potential role of one of hypoxia-induced lncRNA, hypoxia-inducible factor 1A antisense RNA 2 (HIF1A-AS2) as a potential biomarker for AMI.

HIF1A-AS2 is the antisense transcript of hypoxia-inducible factor 1α (HIF1α). It is localized at chromosome 14q23.2 and was first discovered in 1990 to be abnormally expressed in clear cell renal carcinoma. It binds in a complementary manner to the 3' untranslated region (3'UTR) of HIF1α mRNA [12]. In 2002, HIF1A-AS2 was found to be expressed in several human tissues, both physiologically and when the tissues had become cancerous [13]. These observations prompted further investigation into HIF1A-AS2. These studies revealed that HIF1A-AS2 was upregulated in various tumors. Therefore, HIF1A-AS2 was identified as a potential biomarker in oncology [14].

HIF1A-AS2 was also found to be hypoxia-inducible and negatively regulates HIF-1a mRNA expression [15]. HIF-1a is a crucial transcription factor that controls oxygen delivery and utilization as a key modulator of oxygen homeostasis. It controls the expression of genes involved in vascular remodeling and angiogenesis, an essential component of the heart's response to ischemia. Consequently, it was suggested that HIF-1a play a critical role in the pathophysiology of ischemic heart disease and heart failure [16, 17].

## Subjects and methods

### Subjects

After receiving the approval of the ethics committee, the current study was carried out on 98 subjects. Forty-eight patients were admitted to the Cardiology Department at Alexandria main University Hospital, suffering from AMI diagnosed based on WHO criteria, excluding patients with a history of old infarction, previous myocardial intervention, and malignant tumor. Fifty healthy volunteers of matching ages and sex served as a control group. All participants were subjected to full history taking, physical examination, and laboratory investigations (hs Troponin I, CBC, hs CRP, Lipid profile). Six months follow up was done to assass the prognostic value of HIF1A-AS2. Relative quantification of HIF1A-AS2 expression was done using real‐time reverse transcription–polymerase chain reaction.

### Relative quantification of HIF 1A ANTISENSE LONG NON-CODING RNA 2 (HIF1A‐AS2) expression

#### RNA extraction

Genomic RNA was extracted from peripheral blood cells using RNeasy mini kit according to manufacturer instructions (QIAGEN, Germany). The concentration and purity of RNA were measured at 260& 280 &230 nm using NanoDrop 2000c Spectrophotometer (Thermo Scientific, USA)**.** Total RNA was kept at -80 °C until further analysis.

#### Complementary DNA (cDNA) synthesis

First, cDNA was synthesized from purified RNA using High-Capacity cDNA Reverse Transcription Kits (Thermo Scientific, USA) according to the manufacturer's protocol. In a volume of 20 µL, 1 µg of RNA was added to the reverse transcription (RT) reaction mix. The prepared reaction mix was incubated in Arktik thermocycler at 25 °C for 10 min, 37 °C for 120 min, and then 85 °C for 5 min.

#### Quantitative real-time (qPCR)

Relative quantification of HIF1A-AS2 was performed using Maxima SYBR Green/ROX qPCR Master Mix (Thermo Scientific, USA), custom made primers (10 pmol/µL), and cDNA in a total volume of 25 µL according to the manufacturer's instructions. Thermal cycling was done using Rotor gene Q real time PCR system (Qiagen, Germany) and included initial denaturation at 95 °C for 10 min followed by 40 cycles of denaturation at 95 °C for 15 s and final annealing and extension at 56 °C for 1 min. Acquisition of SYBR Green I dye was done during the annealing-extension step. A negative control (No template control) was included in each run. A normalizer target, SF3a1, was used as a housekeeping gene, and relative quantification of HIF1A-AS2 was measured using the Comparative C_T_ method (2^−ΔΔCt^). Primers' sequences are illustrated in Table 1

**Table 1:** PCR primers

	**NCBI Accession Number**	**Forward Primer**	**Reverse Primer**
HIF1A-AS2	NR_045406	GGTCTGCCATCTATTACTT	TCTCAGCATTATAGTCACAA
SF3a1	NM_005877	GATTGGCCCCAGCAAGCC	TGCGGAGACAACTGTAGTACG

### Statistical analysis

Data were analyzed using Statistical Package of Social Science (SPSS/version 23) software. Qualitative data were described using numbers and percentages and were compared using the Chi-square test. The Kolmogorov–Smirnov test was used to verify the normality of the distribution of variables. The student's t-test was used to compare normally distributed quantitative data, expressed in mean ± standard deviation (SD). Mann–Whitney test was used to compare abnormally distributed quantitative variables expressed in the median, minimum, and maximum. Correlations between two quantitative variables were assessed using the Pearson coefficient. The HIF1A-AS2 Receiver operating characteristic curve (ROC) was used to detect diagnostic performance. The significance of the obtained results was determined at the 5% level.

## Results

### Characteristics of the study participants

The current case–control study was conducted on 48 patients diagnosed with AMI according to WHO criteria, with ages ranging from 30.0—75.0 years. Patients included 32 males (66.7%) and 16 females (33.3%). Concerning risk factors, 23 patients were smokers, 16 patients were diabetics, and 27 patients had hypertension. According to ECG changes, 27 patients were diagnosed with Acute ST-segment–elevation MI (STEMI) and 21 with Non-STEMI (NSTEMI). After 6 months of follow up, echocardiography revealed that ejection fraction (EF) in 18 patients was (EF) ≤ 40%, while EF was > 40% in 30 patients. Age and gender were not significantly different between AMI patients and controls (*P* = 0.07 and *P* = 0.14, respectively). Demographic, laboratory, and clinical data for the study groups are illustrated in Table 2.

**Table 2:** Demographic, clinical and laboratory data for the studied groups

	**Acute myocardial infarction Cases** **(*n* = 48)**	**Controls** **(*n* = 50)**	**Test of Sig**	***P***
**Age (year)**				
Mean ± SD	56.23 ± 10.34	52.70 ± 8.74	t = 1.828	0.071
Min. – Max	30.0—75.0	30.0—69.0		
**Sex**				
Male	32 (66.7%)	26 (52.0%)	χ^2^ = 2.181	0.140
Female	16 (33.3%)	24 (48.0%)		
**Type of AMI**				
NSTEMI	21 (43.8%)	-	-	-
STEMI	27 (56.3%)	-		
**Smoking**				
Nonsmoker	22 (45.8%)	30 (60.0%)	χ^2^ = 3.351	^MC^*p* = 0.187
Smoker	23 (47.9%)	15 (30.0%)		
Ex-smoker	3 (6.3%)	5 (10.0%)		
**Diabetes Mellitus**				
Yes	16 (33.3%)	-		
NO	32 (66.7%)			
**Hypertension**				
Yes	27 (56.3%)	-		
No	21 (43.7%)			
**hs- cTnI (ng/ml)**				
Mean ± SD	64.43 ± 109.63	-	-	-
Median (Min. – Max.)	30.04 (1.03–450.0)			
**WBCs (× 10**^**3**^**/ul)**				
Mean ± SD	12.45 ± 5.62	-	-	-
Median (Min. – Max.)	11.18 (3.65–27.80)			
**CRP (mg/dl)**				
Mean ± SD	90.38 ± 84.99	-	-	-
Median (Min. – Max.)	63.0 (1.36—325.0)			
**Ejection fraction**				
≤ 40	18(37.5%)			
> 40	30(62.5%)			

*SD* Standard deviation, *STEMI* ST-segment–elevation MI, *NSTEMI* Non-STEMI, χ^2^ Chi square test, *t* Student t-test

### Expression of HIF1A-AS2 in acute myocardial infarction

**Fig. 1:**
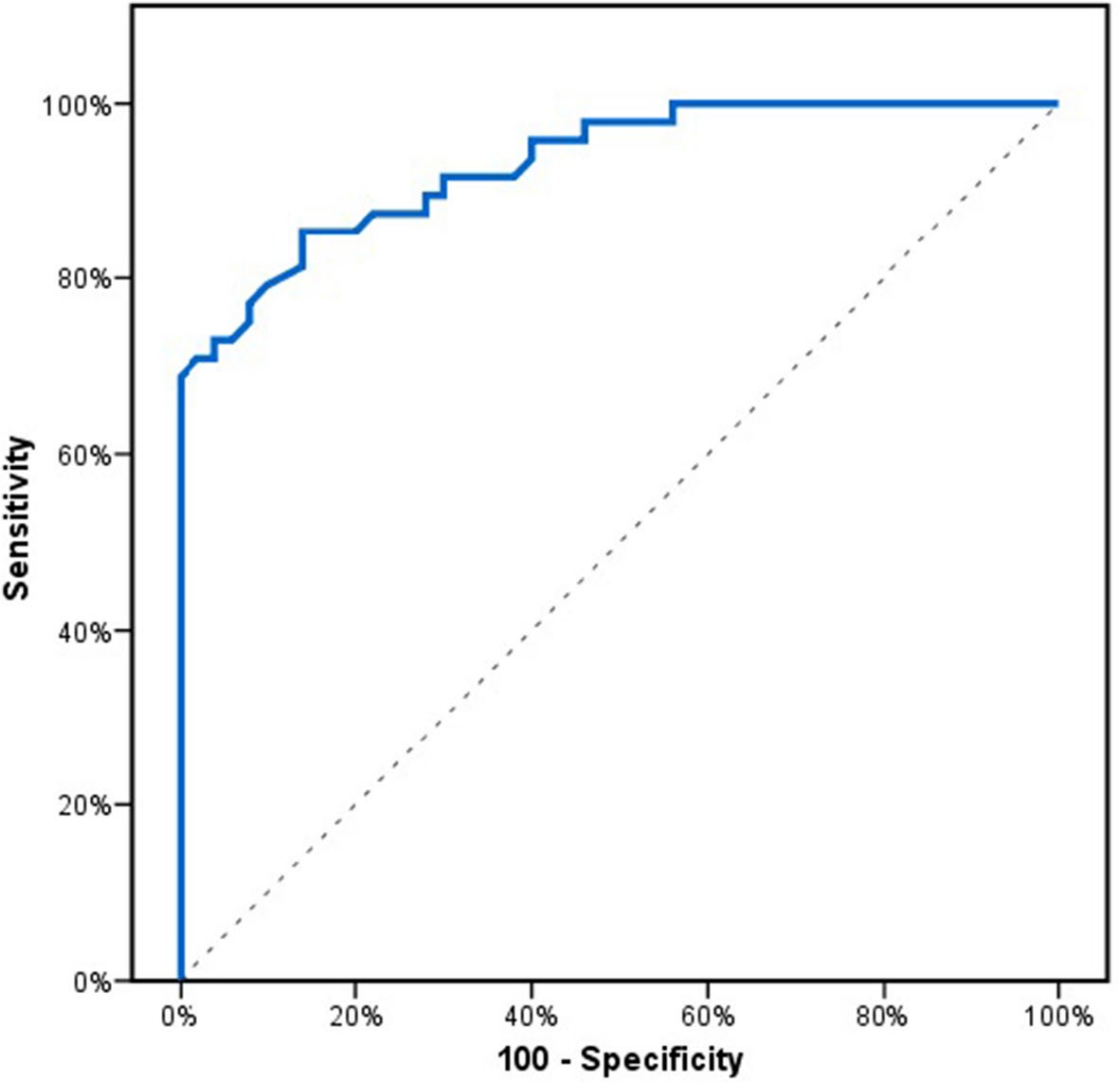
ROC analysis to assess the diagnostic performance of HIF1A-AS2 expression in acute myocardial infarction

The HIF1A‐AS2 expression level was significantly higher in AMI patients than in controls (*P* ≤ 0.001) (Table 3). A ROC curve analysis was done to assess the diagnostic value of HIF1A-AS2 expression in AMI. It was found that HIF1A-AS2 could detect AMI in the patient group with 85.4% sensitivity and 86% specificity at cut-off 1.8. The corresponding area under the curve (AUC) was found to be 0.931 (Fig. 1).

**Table 3:** HIF1A-AS2 expression in acute myocardial infarction

HIF1A‐AS2 expression	Acute myocardial infarction Cases (*n* = 48)	Controls (*n* = 50)	Test of Sig	*P*
Median (Min. – Max.)	5.75 (0.99—310.0)	1.07 (0.50—2.60)	U = 164.50^*^	< 0.001^*^

*U* Mann Whitney test

^*^Statistically significant at *p* ≤ 0.05

### Assessment of HIF1A-AS2 value as an early AMI biomarker

Interestingly, patients presenting within three hours showed significantly higher HIF1A‐AS2 expression levels than patients presenting later (*P* = 0.002) (Table 4).

**Table 4:** Association between HIF1A‐AS2 expression levels and timing of presentation

	**Onset of chest pain**		**(*P*-value)**
	** ≤ 3 Hour** **(*N* = 17)**	** > 3 h** **(*N* = 31)**	
**HIF1A-AS2 expression**			
Median	28.2	3.1	0.002^*^
Min.—Max	1.6 – 265.3	0.99 – 310.0	

*p p* values for Mann Whitney test for comparing between the two groups

^*^Statistically significant at *p* ≤ 0.05

**Fig. 2:**
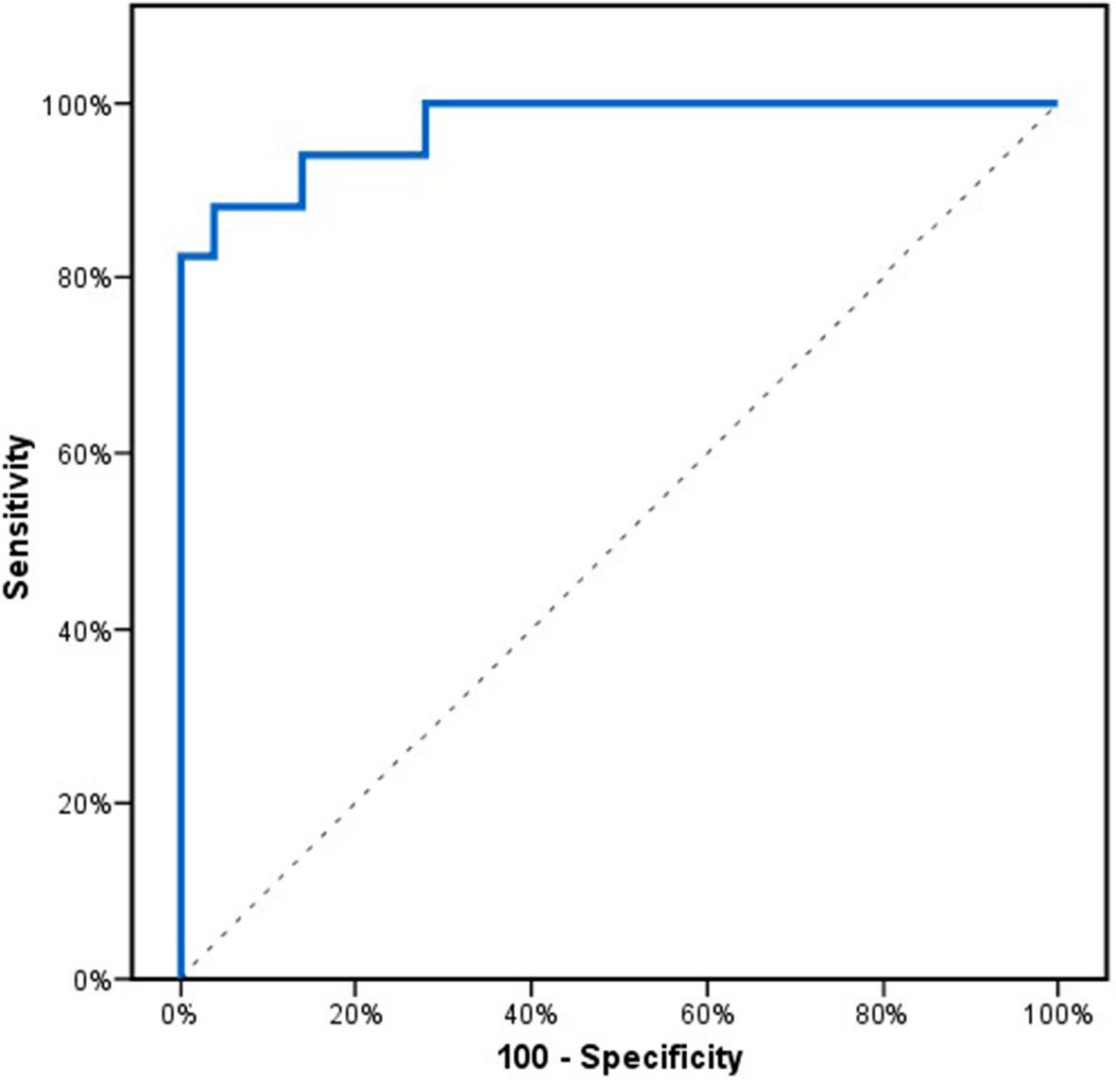
ROC analysis to assess the diagnostic performance of HIF1A-AS2 expression as an early marker for acute myocardial infarction

In order to determine the role of HIF1A-AS2 as an early marker for AMI, another ROC curve for HIF1A-AS2 expression was constructed to detect AMI in the patient group with pain onset within three hours vs. the control group. We found that HIF1A-AS2 can detect early MI with 94% sensitivity and 86% specificity at a cut-off > 1.8 (Fig. 2).

### Associations between HIF1A‐AS2 expression and clinical and laboratory parameters in acute myocardial infarction patients (Table 5)

**Table 5:** Associations between HIF1A‐AS2 expression and clinical and laboratory parameters in acute myocardial infarction patients (*n* = 48)

	**HIF1A‐AS2 expression**	**Test of Sig**	**P**
	**Median (Min. – Max.)**		
**Sex**			
Male	5.70 (1.27- 310.0)	U = 252.0	0.930
Female	6.01 (0.99 – 132.0)		
**Type**			
NSTEMI	6.70 (0.99–265.32)	U = 255.0	0.554
STEMI	4.70 (1.14–310.0)		
**Smoking**			
Nonsmoker	7.9 (1.0 – 265.3)	H = 2.322	0.313
Smoker	4.6 (1.3 – 114.7)		
Ex-smoker	3.1 (1.6 – 310.0)		
**Diabetes Mellitus**			
No	6.02 (1.27—180.0)	U = 252.5	0.939
Yes	4.7 (1.0 – 310.0)		
**Hypertension**			
No	3.1 (1.3 – 114.7)	U = 212.50	0.140
Yes	6.7 (1.0 – 310.0)		
**Ejection fraction**			
≤ 40	3.1 (0.99 – 28.2)	U = 149.5^*^	0.001*
> 40	63.8 (2.6 – 310.0)		

*U* Mann Whitney test, *H* H for Kruskal Wallis test

^*^ Statistically significant at *p* ≤ 0.05

There was no correlation between the expression of the HIF1A-AS2 gene and age (*P* = 0.639). In addition, no significant association was found between HIF1A-AS2 and gender or any AMI risk factors such as smoking, hypertension, and diabetes mellitus. In addition, no significant difference in HIF1A-AS2 levels between STEMI and NSTEMI patients was detected.

HIF1A-AS2 demonstrated positive correlation with high sensitivity cTnI level (*r* = 0.437, *p* = 0.002). With respect to markers of inflammation, positive correlation was found between HIF1A-AS2 and CRP level (*r* = 0.42, *P* = 0.002) and WBC count (*r* = 318, *P* = 0.028).

### HIF1A‐AS2 expression as a predictor of left ventricular dysfunction

**Fig. 3:**
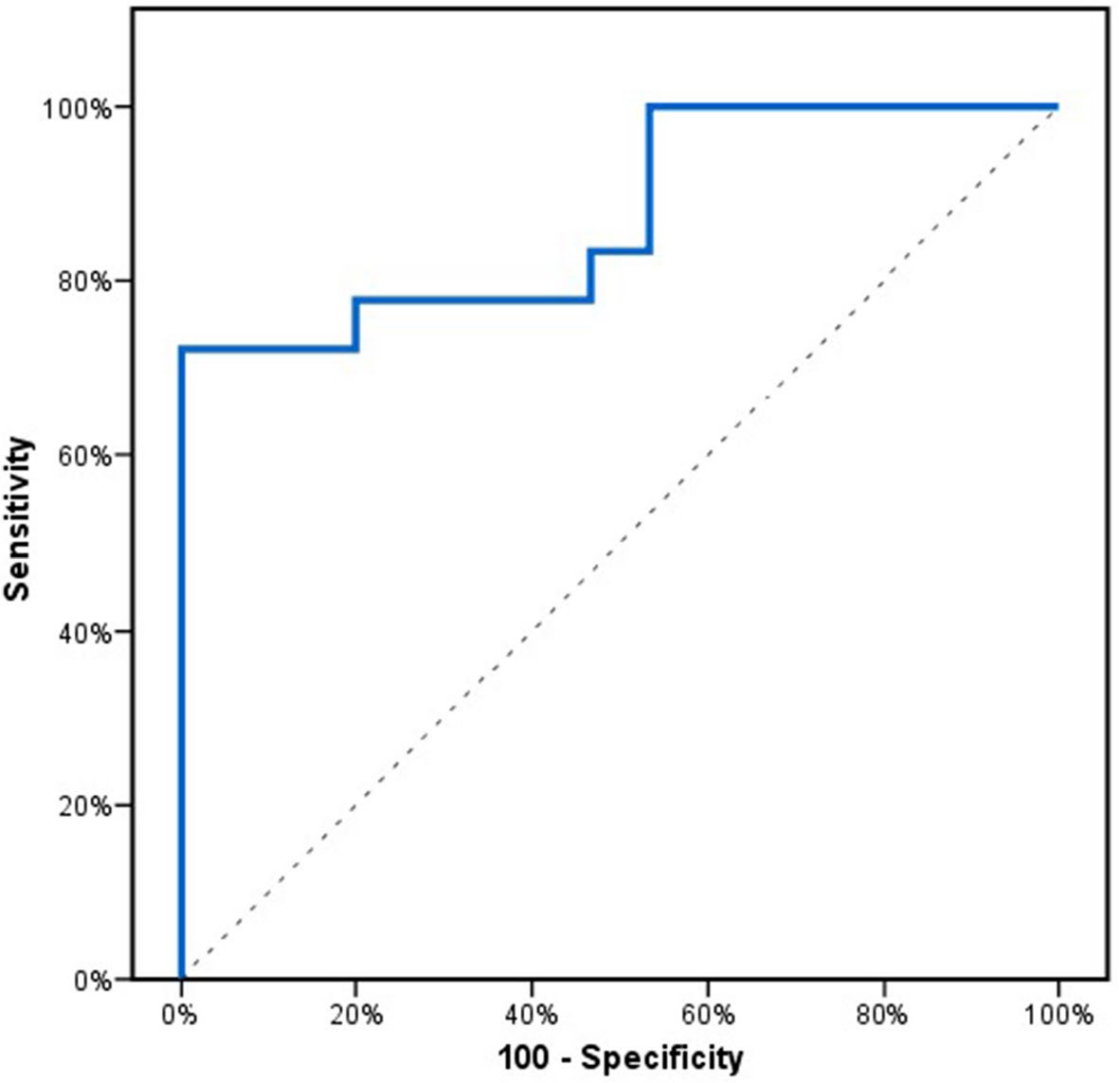
ROC analysis to assess the performance of HIF1A-AS2 expression as a predictor of left ventricular dysfunction in acute myocardial infarction

In the patient group, a significant association was found between HIF1A-AS2 and EF% after 6 months of follow up (*p* = 0.001). The overexpression of HIF1A-AS2 using multivariate regression analysis was found to be still significantly associated with low EF (*P* = 0.018, OR 1.133). The ROC curve of HIF1A-AS2 expression was constructed to predict post-MI left ventricular (LV) dysfunction (EF% < 40) in the patient group. The corresponding area under the curve (AUC) was found to be 0.874. The best cut-off value for HIF1A-AS2 when predicting post-MI LV dysfunction in this patient group was fold change > 6.7, where sensitivity = 77.8% and specificity = 80.0% (Fig. 3).

## Discussion

MI is one of the most prevalent cardiovascular diseases characterized by a disruption in the blood and oxygen supply to the heart, leading to gradual myocardial necrosis and heart failure [18]. Therefore, the identification of an ideal biomarker for early detection of MI can contribute to improving MI diagnosis and prognosis.

In the last two decades, epigenetics has emerged as a regulatory interface between the environmental factors and genome with remarkable advances in high throughput' omics' technologies. Due to the limited proliferative ability of human cardiomyocytes, epigenetic regulation appears to play a particularly crucial role in the heart. It permits the heart to respond quickly and adequately to environmental challenges and cardiac stress [19].

Although lncRNAs that control gene expression through diverse mechanisms have been primarily associated with cancers [14], it is now evident that various lncRNAs play a vital role in cardiac development and function. Their dysregulation is involved in the onset and progression of various cardiovascular diseases [20]. It has been found that some lncRNAs are modulators in hypoxia, acting as stimulators or inhibitors of the HIF-pathway and thus called hypoxia responsive lncRNAs [21].

HIF1A-AS2, the antisense lncRNA transcribed from HIF-1a mRNA, has been identified in cancer processes associated with hypoxia. Consequently, it is proposed to be involved in physiological and pathological processes in ischemic and hypoxic conditions [22]. Two previous studies have suggested that HIF1A‐AS2 is overexpressed in MI and coronary heart disease [23, 24], highlighting its potential as a MI biomarker and therapeutic target. However, its role as an early diagnostic and prognostic marker remains unclear.

The current study was designed to determine the clinical significance of HIF1A-AS2 in AMI as a biomarker for early diagnosis (in the first 3 h from pain onset) and prognosis. It was found that there was a highly significant increase in the levels of HIF1A-AS2 expression in MI patients when compared to the control group with 85.4% sensitivity and 86% specificity at a cut-off value > 1.8. Interestingly, we found that the HIF1A-AS2 level is substantially higher in the first 3 h from the onset of chest pain than later, and when compared with its level in controls, we found that it has a higher sensitivity (94%) and the same specificity as an early biomarker.

According to previous research, the HIF1A-AS2 promoter shows several putative hypoxia response elements (HRE), which can explain the overexpression of HIF1A-AS2 under hypoxic conditions present in AMI. In addition, it was found that HIF1A-AS2 could expose AU-rich elements present in the 3’ untranslated region of HIF-1a mRNA, thereby destabilizing HIF-1A mRNA and thus possibly increasing the degradation of HIF-1a mRNA, inducing a negative loop of regulation of HIF-1a expression [10].

In another study, HIF1A-AS2 was found to be overexpressed in human umbilical vein endothelial cells (HUVECs) in hypoxia. However, Luciferase reporter assay revealed that HIF1A-AS2 upregulates HIF-1α by sponging miR-153-3p, which is responsible for posttranscriptional silencing of HIF-1α. HIF1A-AS2 was suggested to promote angiogenesis in HUVECs in hypoxia by increasing HIF-1 α expression [22].

In the current study, HIF1A‐AS2 was positively correlated with inflammatory markers such as CRP and WBCs count in the patient group. Vausort et al. also found positive associations between HIF1A‐AS2 and several inflammatroy markers measured at admission, including CRP, white blood cell count, and percentage of neutrophils. They suggested that the increased expression of lncRNA is not due to hypoxia, but rather the inflammatory response to MI. They measured the expression of HIF1A‐AS2 in different blood cells from healthy donors and found that monocytes and neutrophils are the primary sources of HIF1A‐AS2. In addition, they revealed a strong positive association between HIF1A‐AS2 and the percentage of circulating neutrophils. Therefore, they suggested that the upregulation of HIF1A‐AS2 in patients with MI is mostly because of neutrophils [23], which may explain why HIF1A‐AS2 was significantly higher in patients who presented within 3 h of chest pain onset than patients who later presented a phase where mainly neutrophils become activated.

The association between HIF1A-AS2 and inflammation was also proposed in another study. They found that HIF1A-AS2 elevates the expression of activating transcription factor 2 (ATF2). ATF2 is expressed in a variety of tissues and involved in inflammatory responses. HIF1A-AS2 also was found to be highly expressed in atherosclerotic mice. Downregulation of HIF1A-AS2 inhibited inflammation by suppressing the levels of pro-inflammatory factors and adhesion molecules. Therefore, HIF1A-AS2 was proposed as a promising biomarker for coronary artery disease (CAD) and a therapeutic target for atherosclerosis [25].

We found a positive correlation between HIF1A-AS2 and known specific cardiac markers in the patient group, specifically with troponin. As reported, troponin is correlated with infarct size and severity [26]. Therefore, there may be a potential association between HIF1A‐AS2 and infarct severity.

In the current study, no significant correlation was found between HIF1A‐AS2 and age. In addition, no significant association was detected between HIF1A‐AS2 and gender, type of MI or any risk factors for MI as with smoking, diabetes mellitus, hypertension, among patient groups. This finding agrees with Vausort. et al. and Zhang. et al. concluded that HIF1A‐AS2 is not affected by age, gender, smoking, diabetes mellitus, or hypertension [23, 24].

Subsequently, we investigated whether HIF1A-AS1 could be used as a predictor for LV dysfunction after 6 months follow up. We found that HIF1A-AS1 levels can be used as a predictor for LV dysfunction after using multivariate logistic regression analysis with 77.8% sensitivity and 80.0% specificity.

The only available data about the role of HIF1A-AS1 as a predictor for post-MI heart failure is from the Vausort V. et al. study. However, they found no association between HIF1A-AS1 and LV dysfunction at a 4-month follow-up, as demonstrated by EF ≤ 40%.

The effect of HIF1A-AS1 overexpression on myocardial apoptosis has been experimentally studied to reveal a novel molecular-targeted therapy in MI. Luo et al. found that the HIF1A-AS2 level is upregulated in hypoxia-treated human cardiomyocytes (HMCs) compared with normal cardiomyocytes. Through increased TRIM44 expression, HIF1A‐AS2 inhibits the Akt pathway via sponging miR‐623 to promote apoptosis of cardiomyocytes. In contrast, HIF1A‐AS2 silencing led to the repression of cardiomyocyte apoptosis and an increase in cardiomyocyte viability, migration, and invasion. These findings propose the promoting effect of HIF1A‐AS2 in MI injury induced by hypoxia and underscore the potential role of HIF1A‐AS2 in targeted treatment for MI patients [27].

Early diagnosis as well as rapid treatment with targeted drugs are both crucial in the management of myocardial infarction patients. Example for targeted druges in myocardial infarction is alirocumab, which is proprotein convertase subtilisin kexin type 9 (PCSK9) inhibitors. PSCK9 was discovered to be associated with elevated low-denisity lipoprotein (LDL) and adverse cardiovascular outcomes. It was observed that among acute myocardial infarction patients, the early addition of alirocumab, to statin therapy resulted in greater coronary atherosclerotic plaque regression in non–infarct-related arteries with subsequent improvement of patients' management, treatment adherence, and quality of life [28–30].

Based on the previous findings, we may conclude that HIF1A‐AS2 might be used as a potential diagnostic biomarker for early MI as well as a predictor for post-MI heart failure. Furthermore, it paves the way for further research to investigate its value as a therapeutic target for MI and heart failure.

We recommend to study the diagnostic performance of HIF1A-AS2 in different clinical settings and comorbidities using larger number of patients. Extended multicentric studies is also recommended to assess the prognostic value of HIF1A-AS2 as a predictor of late ventricular dysfunction in the initial evaluation of the patients. This may be useful in choosing the revascularization strategy to be initially adopted in a STEMI patient, or in deciding about the urgency of an invasive evaluation in the face of a patient without ST-segment elevation MI.

## Data Availability

The data supporting the conclusions are included within the article.

## Abbreviations

AMI: Acute myocardial infarction
lncRNAs: Long non-coding RNAs
HIF1A-AS2: Hypoxia-inducible factor 1A antisense RNA 2
LVD: Left ventricular dysfunction
IHD: Ischemic heart disease
STEMI: ST-segment–elevation MI
ECG: Electrocardiographic
HIF1α: Hypoxia-inducible factor 1α
qPCR: Quantitative real-time PCR
SD: Standard deviation
ROC: Receiver operating characteristic curve
EF: Ejection fraction
AUC: Area under the curve
HUVECs: Human umbilical vein endothelial cells
WBC: White blood cells count
CRP: C- reactive protein
ATF2: Activating transcription factor 2
HMCs: Hypoxia-treated human cardiomyocytes
